# HHV-6A Infection and Systemic Sclerosis: Clues of a Possible Association

**DOI:** 10.3390/microorganisms8010039

**Published:** 2019-12-24

**Authors:** Elisabetta Caselli, Irene Soffritti, Maria D’Accolti, Daria Bortolotti, Roberta Rizzo, Gianluca Sighinolfi, Dilia Giuggioli, Clodoveo Ferri

**Affiliations:** 1 Section of Microbiology and Medical Genetics, Department of Chemical and Pharmaceutical Sciences, University of Ferrara, 44121 Ferrara, Italy; 2 Rheumatology Unit, Medical School, University of Modena and Reggio Emilia, University-Hospital Policlinico of Modena, 41121 Modena, Italy

**Keywords:** systemic sclerosis, HHV-6, immune response, HLA-G

## Abstract

Systemic sclerosis (SSc) is an autoimmune disease characterized by vasculopathy, excessive extracellular matrix deposition, and fibrosis of the skin and internal organs. Several infectious agents, including human herpesvirus-6 (HHV-6), have been suggested as possible triggering factors, but a direct association is still missing. We characterized 26 SSc patients for the presence of HHV-6 in tissues and blood, the anti-HHV-6 response, HLA-G plasma levels, and KIR typing. Given the prominent role of endothelial cells (EC) in SSc pathogenesis, along with HHV-6 tropism for EC, we also investigated the expression of pro-fibrosis factors in HHV-6 infected EC. Results showed the presence of HHV-6A in skin biopsies, and an increased virus load was associated with disease severity and poor natural killer (NK) response against the virus, particularly in subjects exhibiting a KIR2 phenotype. HLA-G plasma levels were significantly higher in HHV-6A/B-KIR2 positive SSc patients and in vitro HHV-6A infection-induced pro-fibrosis factors expression in EC, supporting its role in the development of the fibrosing process. Our data suggest an association between virus infection/reactivation and disease, opening the way to future studies to understand the mechanisms by which HHV-6A might contribute to the multifactorial pathogenesis of SSc.

## 1. Introduction

Systemic sclerosis (SSc) is a rare autoimmune disease representing one of the most severe connective tissue pathologies, characterized by vascular obliteration, immunological abnormalities, and excessive extracellular matrix deposition, which causes fibrosis of the skin and of internal organs [1]. The etiopathogenesis of the disease is still unknown; possibly, multiple initial events may trigger the release of different cytokines by inflammatory cells, diffuse microangiopathy, fibroblast activation with abnormal collagen deposition, and subsequent ischemic and fibrotic manifestations [2].

The disease is likely a multistep and multifactorial process involving immune system alterations, genetic, and/or environmental factors [1,3,4]; in this scenario, several infectious agents (both bacterial and viral) have been suggested as possible triggering factors, including Parvovirus B19, human Cytomegalovirus (CMV), Epstein Barr virus (EBV), and human herpesvirus 6 (HHV-6) [5]. 

HHV-6 infection is common and has a worldwide distribution [6]. The virus belongs to the β Herpesvirinae subfamily, and two species are recognized, HHV-6A and HHV-6B, which share high genome similarity but differ for some biologic properties, epidemiology, and disease association [7]. Although they are classified as lymphotropic viruses, their in vivo tropism is considerably broader, including T-lymphocytes, macrophages, endothelial cells (ECs), salivary glands, and brain [6,8,9,10,11], thyroid epithelial cells [12], natural killer (NK) cells [13], and endometrial cells [14]. 

After primary infection, the virus establishes a latent infection, residing mainly in peripheral blood mononuclear cells (PBMCs) and macrophages [15,16], and expressing a few specific viral transcripts including U94, which in the absence of other viral lytic transcripts is considered a molecular marker of viral latency [17,18].

As to the pathogenic associations, HHV-6B is the recognized etiologic agent of roseola (*Exanthem subitum*), a childhood benign febrile disease, whereas HHV-6A has not yet clearly been associated with a specific disease [19] but seems to be involved in many autoimmune diseases, especially during immune dysregulation [20]. Virus-associated pathologies, where virus reactivation has been detected [21,22], include autoimmune thyroiditis [12], multiple sclerosis [23,24,25], Sjogren syndrome [26,27], rheumatoid arthritis and systemic lupus erythematosus [28,29], *Purpura fulminans* and severe autoimmune acquired protein S deficiency [30], and severe autoimmune hepatitis [31]. However, the possible role of HHV-6A/B infection and/or reactivation in the development of SSc is still debated.

On the immunological side, the killer immunoglobulin-like receptor KIR2DL2 has been recently associated with a higher risk to develop SSc [32], but it is also recognized as a factor impairing the anti-herpesviral immune response, favoring herpesvirus infection [33,34,35], and we observed a consistent impaired NK response against HHV-6 in multiple sclerosis patients expressing KIR2DL2 type [25]. In parallel, we also reported that HHV-6A/B infection modulates the expression of the tolerogenic human leucocyte antigen (HLA)-G in different cell types [36,37], and this molecule was also reported to be differentially expressed in SSc patients compared to controls, both at the skin and blood level [38,39].

Based on these observations, this study explored the possible association between HHV-6A/B infection and SSc development, including different sides of the hypothesis in the analysis. We investigated the presence and replicative state of HHV-6A and 6B in SSc patients both at the blood and tissue level, the immune responses against the virus (focusing in particular on antibody and NK response), the host KIR type and HLA-G expression, and, as a proof of concept of HHV-6A and 6B involvement in fibrosis induction, the ability of HHV-6A/B infection to induce an aberrant expression of pro-fibrotic factors in vascular endothelial cells. Here, we report the results of our studies on the possible role of HHV-6A/B infection in the pathogenesis of SSc.

## 2. Materials and Methods

### 2.1. Study Population

After approval of the study by the local Institutional Review Board and “Area Vasta Emilia Nord” Ethical Committee (Project identification code: 2742016; 27 October 2016), 26 unselected SSc patients (22 women and four men), all referred to the Rheumatology Unit, University-Hospital Policlinico of Modena, Italy, were recruited. All enrolled subjects gave informed consent. The study was conducted in accordance with the Declaration of Helsinki. All patients fulfilled the 2013 ACR/EULAR criteria for SSc and were clinically classified according to the extent of skin involvement in limited and diffuse SSc [40]. The mean age of SSc patients was 56 years (range 38–74 years). Clinico-epidemiological and laboratory investigations including the modified Rodnan skin score (mRSS) to evaluate the extent of skin fibrosis [41,42] and the main visceral organ involvement according to standardized methodologies [1,3]. Thirty healthy subjects were included as controls (25 women and five men; mean age 52, range 38–65). A percentage corresponding to 78.9% of patients and 75% of healthy controls were positive for the presence of anti-HHV6 IgG antibodies, as judged from routine laboratory tests and ELISA assay against HHV6A virus lysate.

### 2.2. Clinical Samples

Heparinized peripheral blood was collected from the enrolled SSc patients and healthy controls. In addition, cutaneous biopsies were obtained from five SSc patients and five healthy controls. 

Peripheral blood mononucleated cells (PBMCs) and plasma samples were obtained by Ficoll density gradient centrifugation (VWR International PBI, Milan, Italy) of 10 mL peripheral blood samples. Aliquots corresponding to 5 × 10^5^ and 10^6^ PBMCs were pelletized and used respectively for DNA and RNA extraction. DNA was extracted by conventional proteinase K-SDS digestion followed by phenol-chloroform purification, as described [43]. Total RNA was extracted by the miRNeasy kit following the manufacturer’s instructions (Qiagen, Hilden, Germany). Elimination of contaminant DNA was assured by DNase digestions and lack of amplification in PCR reactions where retrotranscription (RT) had been omitted [44].

Skin biopsies were frozen in liquid nitrogen immediately after collection and kept at −80 °C until use. Total DNA and RNA were extracted from biopsies respectively by the SDS-proteinase K digestion and phenol-chloroform method and by Fibrous RNeasy kit (Qiagen, Hilden, Germany). Extracted nucleic acids were quantified by Nanodrop spectrophotometric reading at 260/280 nm. 

### 2.3. HHV-6 Serology

The presence of anti-HHV6A/B antibodies was verified at enrollment by a routine laboratory test in all the subjects enrolled in the study. In addition, plasma samples from recruited patients and healthy controls were analyzed for the presence and titer of antibodies against HHV-6A/B virus lysate and the specific U94 virus antigen, using previously set-up ELISA assays [12,45].

### 2.4. Analysis of KIR Type

SSc patients and controls were analyzed for killer-cell immunoglobulin-like (KIR) type, focusing on KIR2DL2 and KIR2DL3 receptors as they have been associated with reduced ability to control herpesvirus infections. Briefly, DNA was extracted as described from PBMCs isolated from SSc subjects and controls, and KIR type was analyzed by specific PCR, as previously described [46].

### 2.5. Virus DNA and RNA Analyses

HHV-6A/B DNA presence was analyzed by a specifically set up digital droplet polymerase chain reaction (ddPCR), specific for the U94 gene using a QX200^TM^ Droplet Digital ^TM^ PCR System (Biorad, Milan, Italy) and the following set of primers and probe: HHV-6 U94(+) (5′-GAG CGC CCG ATA TTA AAT GGA T-3′); HHV-6 U94(-) (5′-GCT TGA GCG TAC CAC TTT GCA-3); HHV-6 U94 TaqMan QSY probe (5′-FAM-CTG GAA TAA TAA AAC TGC CGT CCC CAC C-QSY-3′) (Thermo Fisher Scientific, Milan, Italy). Amplification of the house-keeping human RNase P gene by a commercial ready-to-use assay (Biorad, Milan, Italy) was performed as control and used to normalize virus counts for cell number.

In positive samples, HHV-6 species identification was performed by a ddPCR specifically discriminating 6A and 6B species, as previously described by others [47]. 

HHV-6A/B transcription was assessed by nested PCR and qPCR after retrotranscription (RT-qPCR), determining the presence of lytic (U42) or latent (U94 in the absence of U42) mRNAs, as previously described [12]. 

### 2.6. Cells and Viruses for In Vitro Virus Infection

Human umbilical vein endothelial cells (HUVEC; ATCC CRL-1730), obtained as previously described [11,37], were grown in EGM-2 medium (BioWhittaker, Walkersville, MD, USA) in collagen-coated plates (Biocoat Collagen, BD Biosciences, Bedford, MA, USA). All the experiments were performed on third to fifth passage HUVECs.

HHV-6A (U1102 strain) and HHV-6B (CV strain) stocks were respectively obtained in J-Jhan (6A) and Sup-T1 (6B) T cells as previously described [18] and contained about 10^10^ genome equivalents per ml. The same stock was used for all the experiments of infection in HUVECs. Inactivated viruses, obtained by UV irradiation of the purified inocula for 30 min at 200 mJ/cm^2^, as previously described [48], were used as controls.

### 2.7. Endothelial Cell In Vitro Infection

HUVECs were infected as previously described with HHV-6A (U1102 strain), HHV-6B (CV strain) and HHV-7 (CZ strain) as a control, using a number of genome equivalents per cell corresponding to 10:1 for all the used viruses [11]. Briefly, HUVECs were seeded at optimal density in six-well collagen-coated plates and infected with the cell-free virus inocula for 3 h. Afterward, inocula were removed, and fresh medium was added to the infected cells. Cells were collected 48 h post-infection (h.p.i.), and total RNA was extracted for microarray analysis. Control cells were uninfected or infected with the same amount of genome equivalents per cell of UV-inactivated cell-free virus inocula.

### 2.8. Analysis of the Expression of Fibrosis-Associated Factors

RNA from infected or uninfected HUVECs was extracted by the miRNeasy kit (Qiagen, Hilden, Germany) as already described [44], and analyzed by a qPCR microarray simultaneously detecting 84 factors associated with fibrosis (Qiagen, Hilden, Germany). Results represented up- or down-regulation of factors in infected versus control cells (either uninfected or infected with UV-inactivated viruses), and were expressed as fold-change compared to values detected in control cells, after normalization for six housekeeping genes (βactin, β2microglobulin, GAPDH, HPRT1, RPLP0, and HGDC). Duplicate samples from three independent experiments were analyzed.

### 2.9. Fluorospot Assay for Immune Cell Activation

PBMCs were isolated by gradient centrifugation from peripheral blood of patients, then 4 × 10^6^ PBMCs were seeded in 12-well plates (1 mL/well) and cultured in RPMI supplemented with trophic factors (PHA, IL-15, and IL-2) for 24 h. Then, NK cells were purified from PBMCs through magnetic cell separation (MACS) system (Miltenyi Biotech, Gladbach, Germany). The NK cell purification is an indirect magnetic labeling system for depletion of human T cells, B cells, and myeloid cells from PBMC to enrich untouched NK cells (negative selection). Purification of NK cells was executed according to the manufacturer’s directions. As determined by flow cytometry with CD3-PerCp-Cy5.5, CD56-FITC moAbs (e-Bioscience, Frankfurt, Germany), the procedure resulted in >90% pure NK cells (data non shown). Meanwhile, 96-well plates were coated overnight with HHV-6A/B lysate or purified U94 protein (2 µg/mL). Purified NK cells (2 × 10^5^ cells/100 µL/well) were then assayed for the ability to recognize and being activated by HHV-6A/B and U94, measuring IFNγ and Granzyme B production. The release of IFN-γ and Granzyme B by stimulated NK cells was assayed by Fluorospot assay (Thermo Fisher Scientific, Milan, Italy), following the manufacturer instructions. The measurement was performed by reading fluorescence by the automated microplate reader Infinite M200, Tecan (Austria GmbH, Grodig, Austria) equipped with filters for excitation 490 nm/emission 510 nm (FITC) for 490-reagent detection and excitation 550 nm/emission 570 nm (Cy3) for 550-reagent detection.

### 2.10. Analysis of HLA-G in Patients’ Blood

Plasma samples derived from whole blood after density centrifugation were analyzed for HLA-G presence by ELISA assay, as previously described [49]. Briefly, sHLA-G plasma levels were measured by an enzyme-linked immunosorbent assay (ELISA) in duplicate according to the Essen workshop (Rebmann et al., 2005). Briefly, 96-well plates were coated with the capture antibody MEM-G9 MoAb (Exbio, Praha, Czech Republic) and the presence of sHLA-G was revealed using anti-β2 microglobulin MoAb—HRP as the detection antibody.

### 2.11. Statistical Analyses

Statistical analyses were performed by Mann–Whitney and Fisher tests. Student’s *t*-test with the Bonferroni correction for multiple comparisons was used for comparison between infected and control cells (microarray data). A corrected *p* (*p_c_*) value ≤ 0.05 was considered significant.

## 3. Results

### 3.1. HHV-6 Detection in SSc Patients

The demographic, clinical, and serological features of the 26 SSc patients and healthy control subjects enrolled in the study are summarized in Table 1.

**Figure 1:**
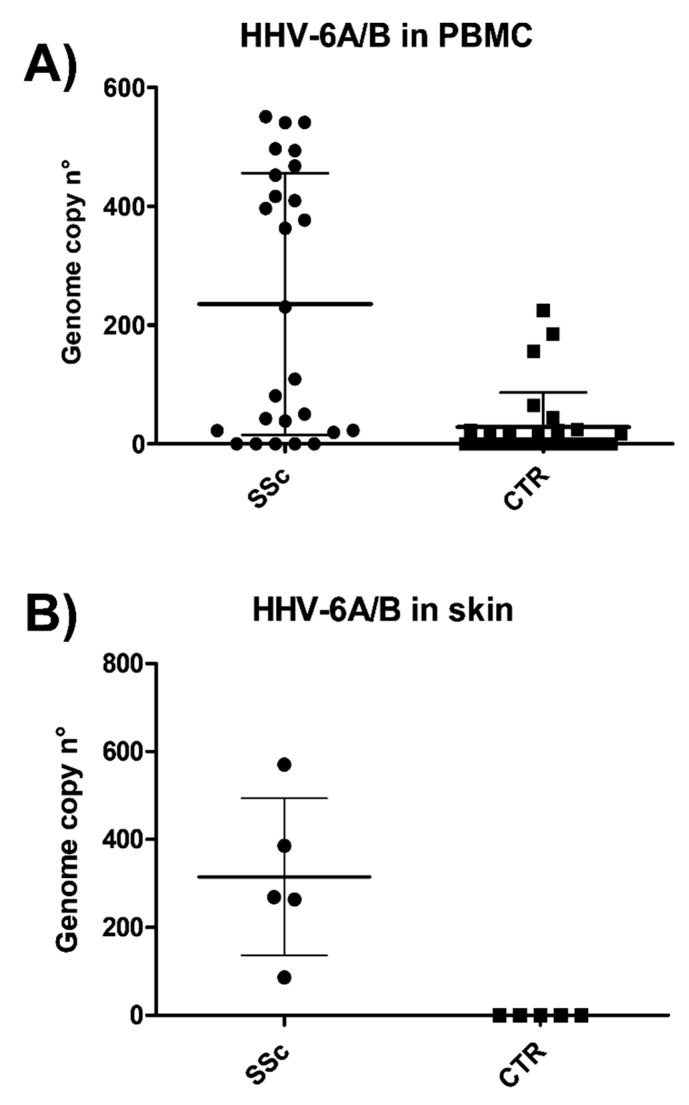
Presence of HHV-6A/B at the blood and skin level in sclerodermic patients and controls. patients. Total DNA extracted from peripheral blood mononuclear cells (PBMCs) (**A**) or skin tissue biopsies (**B**) was extracted and analyzed by a digital droplet polymerase chain reaction (ddPCR) for U94, detecting both virus species. Marks represent the mean values of duplicate samples from each individual; mean line and standard deviation (SD) values are also shown. SSc, systemic sclerosis patients; CTR, controls.

From each subject, peripheral blood was collected and analyzed for the presence of HHV-6A/B, performed by ddPCR designed in the conserved U94 gene and detecting both species. The raw copy number was normalized for the cell number, as detected by the RNaseP copy number, and expressed as the virus genome copy number per 10^6^ cells. The results evidenced that HHV6A or 6B were detectable in a high proportion of PBMCs from SSc patients (21/26, 80.7%) compared to healthy controls (12/30, 40%) (*p* < 0.001) (Figure 1A), and that viral load was significantly higher in the SSc group (median copy number 235.5, range 19–550 per 10^6^ cells) with respect to healthy subjects (mean copy number 18.9, range 18–224 per 10^6^ cells) (*p* < 0.0001). Indeed, as judged by ddPCR results, 61.9% of samples derived from SSc PBMCs had >100 copies of HHV6A/B genomes per 10^6^ cells, whereas only 30% of PBMCs from control group contained >100 copies of virus genomes per 10^6^ cells.

The analysis of HHV-6A/B at the tissue level, performed by ddPCR on skin biopsies, evidenced the presence of virus DNA in all the SSc collected samples, although with different loads (Figure 1B). The mean genome copy number was 315 per 10^6^ cells (range 86–570), and 4/5 samples (80%) contained more than 100 copies/10^6^ cells. No skin samples from healthy controls had detectable levels of HHV-6A/B (*p* < 0.005 by paired *t*-test).

**Figure 2:**
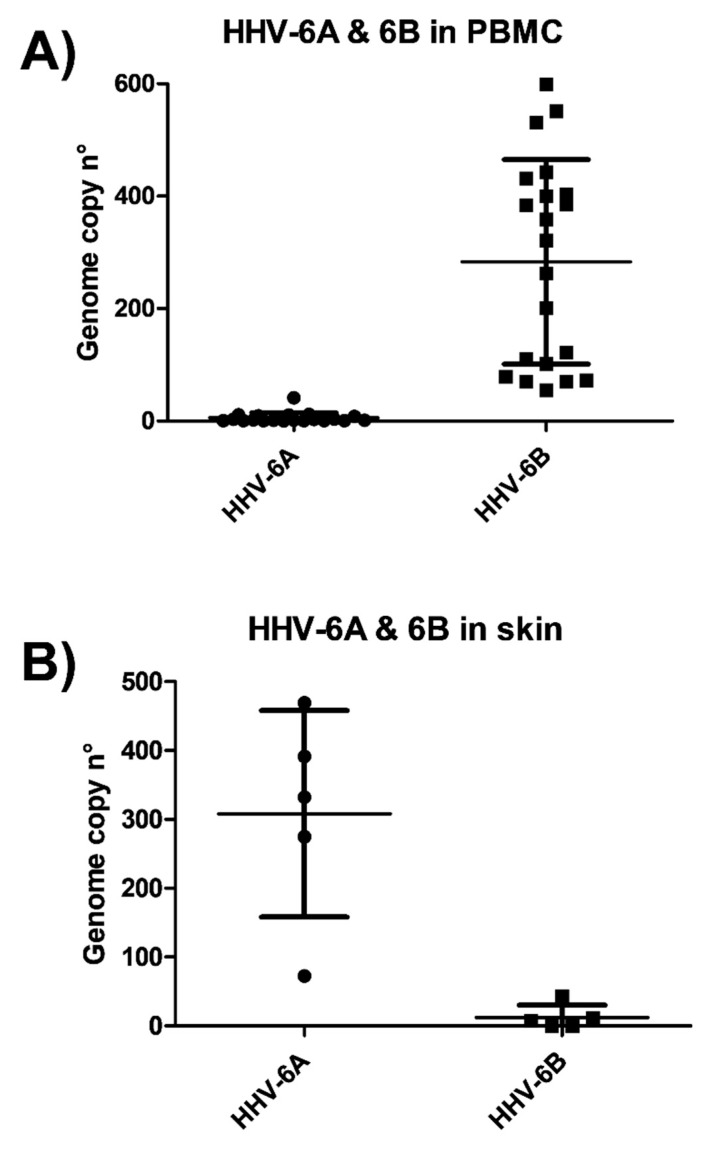
Prevalence of HHV-6A and HHV-6B species at the blood and skin level in SSc patients. Total DNA extracted from skin tissue biopsies (**A**) or PBMC from peripheral blood (**B**) was extracted and analyzed by ddPCR discriminating between the two virus species. Results are expressed as genome copy number per µg of DNA, and represent the mean values of duplicate samples. Mean line and SD values are also shown.

The species identification, performed in each HHV-6A/B positive sample by a specific ddPCR discriminating the two virus species, evidenced that species 6A was prevalent in the skin tissue, although 6B was also detectable at a low level; whereas species 6B was almost exclusively harbored in PBMC samples (Figure 2).

**Figure 3:**
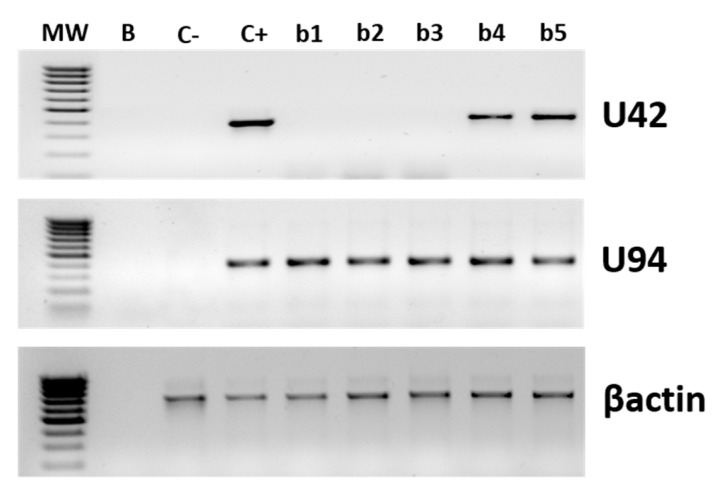
HHV-6A/B replicative state in skin biopsies. Virus transcription was evaluated by nested PCR after retrotranscription, performed on total RNA extracted from skin specimens and PBMCs. Results refer to the amplification of U94 and U42 virus genes, and the human house-keeping β-actin gene as a control. MW, Molecular Weight marker; B, blank; C-, negative control; C+, positive control; b1–5, biopsy 1–5.

Next, we analyzed the virus transcriptional state in the HHV-6A/B positive skin and PBMC specimens. Two virus genes were tested: U94, as the detection of its transcripts, in the absence of other early transcripts, is indicative of latent infection, and U42, as its transcription is indicative of productive infection. The transcriptional analysis, performed by nested PCR and qPCR after retrotranscription (RT-qPCR) on all HHV-6-positive samples, evidenced productive replication in two out of five biopsies (2/5, 40%), where U42 transcripts were detected in addition to U94 ones, although at very low levels (<100 copies per 10^6^ cells) (Figure 3). The two U42-positive biopsies were also those harboring the highest virus load, as detected by ddPCR. By contrast, the analysis of the transcriptional state of the HHV6 in PBMCs evidenced the only transcription of U94 gene in both SSc and control PBMCs, with no concurrent transcription of U42 (used as a marker of productive infection), suggesting that the virus was latent in all samples, at least at the moment of the withdrawal (not shown).

### 3.2. HHV-6 Serology of SSc Patients

**Figure 4:**
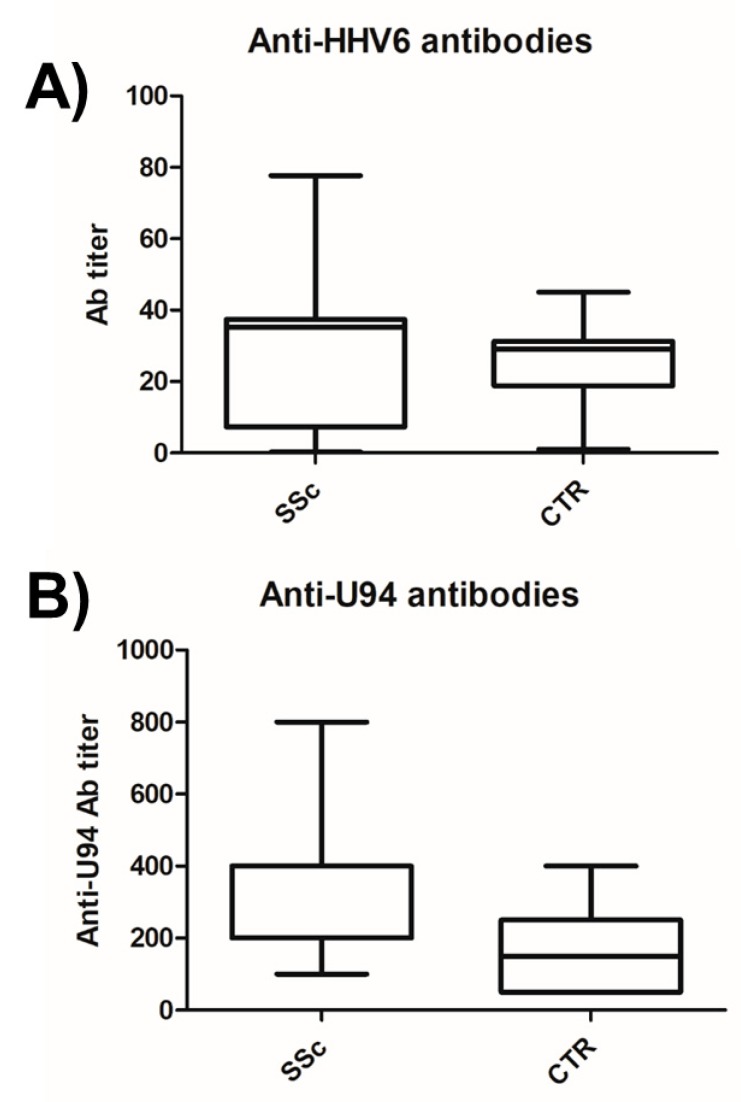
Presence of anti-HHV6A/B antibodies in plasma samples of sclerodermic patients and controls. (**A**) Antibodies directed against the whole virus lysate. (**B**) Antibodies directed against the specific U94 virus antigen. Graphed values correspond to duplicate samples and show median, min, and max values. SSc, systemic sclerosis patients; CTR, controls.

Enrolled SSc patients were characterized for anti-HHV-6 antibody seropositivity by routine laboratory analyses, showing a high prevalence of positive subjects (76.9%), as expected based on the high prevalence of HHV-6A/B infection in the general population. No statistically significant different prevalence or titer was observed in the SSc group compared to the control group (*p* = n.s.), similar to what was reported previously for other autoimmune pathologies (Figure 4A). However, based on previous observations evidencing potentially significant differences in the antibodies specifically directed against the U94 virus antigen, even in the absence of significant differences in the anti-HHV-6A/B antibody prevalence or titer [12,45], we analyzed the antibody response against U94 in the SSc patients cohort (Figure 4B). The results showed both a different prevalence and titer of the anti-U94 antibodies in the SSc group compared to the healthy controls (*p* < 0.004), as the presence of anti-U94 antibodies was detected in 26/26 (100%) of the SSc patients and in 14/30 (46.7%) of the healthy subjects; in addition, their titer was significantly higher in the SSc group (median titer 1:403, range 1:100–800), whereas control group displayed a mean titer corresponding to 1:166 (range 1:50–400), confirming previous results [45].

**Figure 5:**
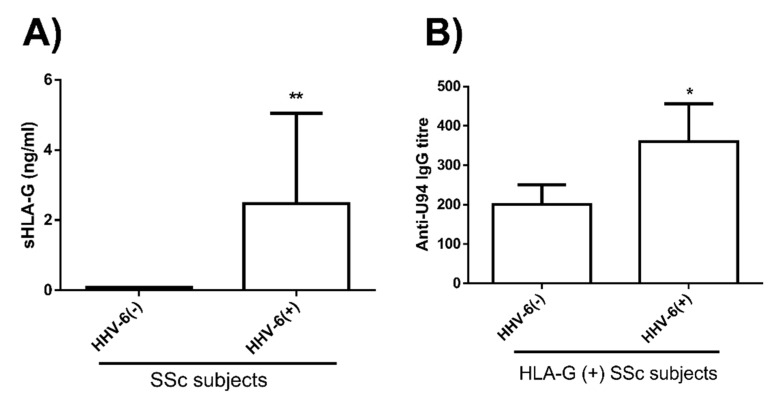
HLA-G plasma levels in SSc patients. (**A**) Level of circulating sHLA-G in plasma of HHV-6A/B negative and positive SSc patients (**, *p* < 0.0001). (**B**) Correlation between HHV-6/HLA-G positivity and anti-U94 antibody titer in SSc patients (*, *p* < 0.05). Results are reported as mean values ± SD.

Since we have already observed that HHV-6A U94 can induce HLA-G in HUVEC cells, inhibiting angiogenesis [37], we analyzed HLA-G plasma levels in correlation with HHV-6A/B presence in SSc patients. As shown in Figure 5, we observed an increase in plasmatic HLA-G in HHV-6 positive patients, compared to HHV-6 negative ones (*p* < 0.0001). Furthermore, the positivity for HLA-G and HHV-6 also correlated with the anti-U94 antibody titer (*p* < 0.05), as HLA-G/HHV-6 positive patients exhibited the highest anti-U94 IgG plasma levels (*p* < 0.05).

### 3.3. KIR Typing in SSc Patients

We previously observed that specific killer-cell immunoglobulin-like (KIR) receptors expressed in natural killer (NK) cells are associated with increased susceptibility to multiple herpesvirus reactivations [25,46]. Thus, SSc patients were characterized for the prevalence of KIR2DL2 and KIR2DL3 types. The results showed no statistically significant differences in the prevalence of DL2 and DL3 types in the SSc group compared to controls, with distribution values in line with data obtained in the general population, although homozygosity for DL2 was slightly higher in our control cohort compared to literature data on healthy population (3.3% vs. 1.2%) [46] (Table 2).

### 3.4. NK Response against HHV-6 in SSc Patients

**Figure 6:**
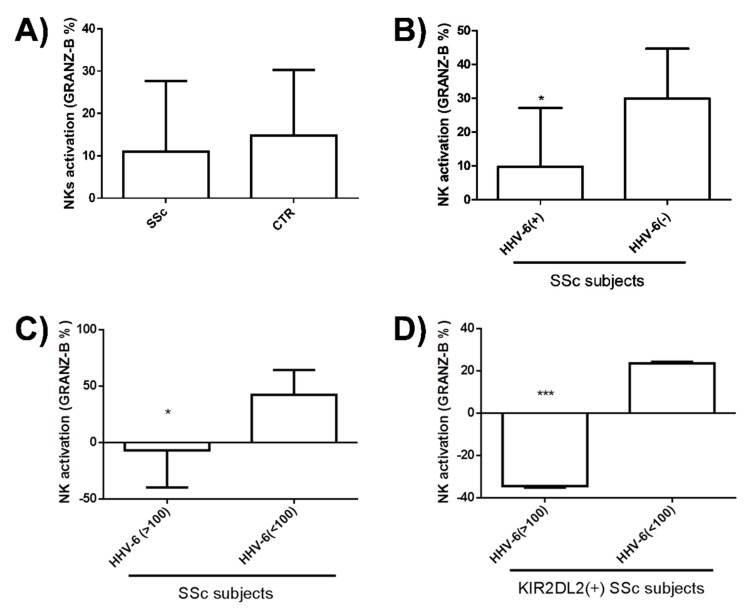
Anti-HHV-6 NK activity in SSc patients. PBMCs isolated from peripheral blood were assayed by Fluorospot for the NK ability to be activated by HHV-6 and U94. (**A**) Natural killer (NK) activation (Granzyme B production) in SSc patients and controls; (**B**) NK activation in SSc patients subdivided for HHV-6A/B positivity or negativity; (**C**) NK activation in HHV-6 positive SSc patients subdivided for HHV-6 load at the tissue level; (**D**) NK activation in SSc patients subdivided for HHV-6A/B skin load and KIR2DL2 presence. Results are expressed as mean value ± SD of duplicate samples (*** *p* < 0.0001; * *p* < 0.05).

NK immune response is crucial for the control of herpesvirus infection. Since we previously observed a defect in NK control in different autoimmune disorders associated with HHV-6A/B infection, we analyzed such a response in SSc patients. In this respect, a Fluorospot assay was performed using NK purified from PBMCs isolated from peripheral blood. Briefly, cultured PBMCs were used to purify NK cells, which were assayed for the ability to recognize and be activated by HHV-6A/B and U94, measuring IFNγ and Granzyme B production. No statistically significant differences were observed between SSc patients and controls with regard to response against virus lysate (not shown). By contrast, as summarized in Figure 6, an impaired NK activity in SSc subjects compared to controls was observed toward the U94 virus antigen, although the difference was not statistically significant (Figure 6A). Notably, the difference was significant when subdividing SSc patients for virus positivity or negativity (as detected by ddPCR), with a clear impairment of NK activity in the HHV-6A/B positive versus negative SSc subjects (Figure 6B). Moreover, among HHV-6A/B positive subjects a statistically significant difference was also observed between groups of SSc patients with a high (>100 copies/µg DNA) or low (<100 copies/µg DNA) virus load at the tissue level (Figure 6C) (*p* < 0.05), with the highest impairment of NK activation observed in SSc subjects positive for virus presence and displaying simultaneously a KIR2DL2 haplotype (*p* < 0.0001) (Figure 6D). By contrast, no significant differences were observed in IFNγ production (data not shown).

### 3.5. HHV-6 Induction of Fibrosis-Associated Factors in Endothelial Cells

**Figure 7:**
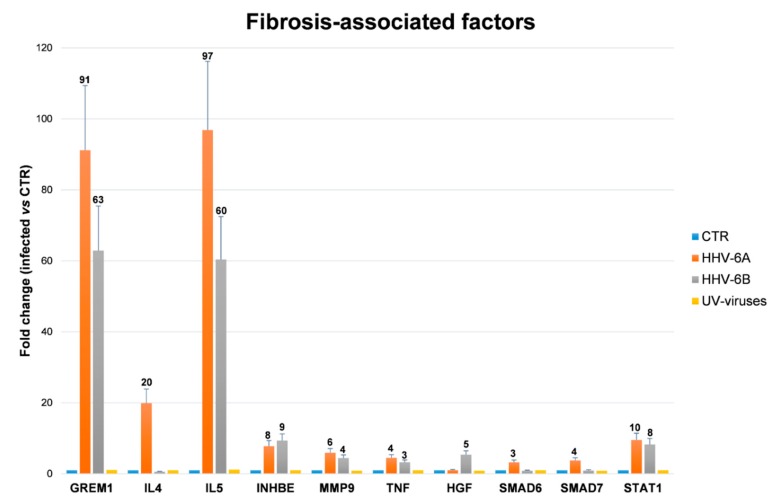
Induction of fibrosis-associated factors by HHV-6A/B infection in human endothelial cells (HUVEC). The modulation of factors potentially associated with fibrosis development was assayed in uninfected HUVEC (CTR) or in HUVEC infected in vitro with HHV-6A, HHV-6B, or the respective UV-inactivated viruses, used as controls. Total RNA was extracted 48 h post-infection and analyzed by a microarray quantifying simultaneously 84 factors associated with fibrosis. Factors modulated by virus infection are shown. Results are expressed as mean values of duplicate samples in three independent experiments ± SD of fold-change compared to values detected in controls (CTR), after normalization for six housekeeping genes (β-actin, β_2_-microglobulin, GAPDH, HPRT1, RPLP0, and HGDC).

Third to fifth passage human primary ECs were infected with cell-free inocula of HHV-6A and HHV-6B, or with the correspondent UV-inactivated viruses. The efficiency of infection was about 50%, and productive replication took place in the first week post-infection, as already previously described [11] (not shown). Analysis of the expression of pro-fibrosis factors, performed by microarray on RNA extracted from infected cells, showed that HHV-6A/B infection can induce the transcription of diverse factors potentially impacting fibrosis development. In particular, after 48 h, 10 factors out of the 84 analyzed appeared to be altered by one or both HHV-6 species (Supplemental Figure S1), whereas no factors were affected by the UV-inactivated viruses used as controls. In detail, both virus species highly up-regulated the expression of the pro-fibrotic factors GREM1 and IL5 (>90 fold by HHV-6A and >60 fold by HHV-6B), and also upregulated to a less extent INHBE, MMP9 and TNF (up to 7.79 fold by HHV-6A and up to 9.35 fold by HHV-6B), while IL4 was upregulated by HHV-6A only (19.89 fold). In parallel, some anti-fibrotic factors were also induced, including SMAD6 and SMAD7 (upregulated only by HHV-6A), STAT1 (induced by both HHV-6A and 6B species), and hepatocyte growth factor (HGF) (by HHV-6B only) (Figure 7). 

## 4. Discussion

Systemic sclerosis (SSc) is a connective tissue disease of unknown etiology, characterized by complex immune system alterations and diffuse microangiopathy and fibrosis of the skin and visceral organs. The etiopathogenesis of SSc may include different environmental factors, among which are microbial infections. In this scenario, infection by HHV-6A/B has been suggested as a potential causative co-factor of the disease, but a definite role has not been established. The same virus has been long associated with different autoimmune conditions involving different cell types and tissues [50], such as autoimmune thyroiditis [12] that is frequently observed in SSc patients. On the other hand, we also reported that the virus can be detected in endothelial cells in vivo [51], and can infect endothelial cells in vitro, exhibiting a potent anti-angiogenetic activity [11].

Based on these observations, our study analyzed virological and immunological footprints of HHV-6A/B infection in SSc patients, also investigating the ability of the virus to induce, in vitro in endothelial cells, the expression of pro-fibrotic factors that could potentially be associated with the development of the disease in vivo.

The results of our investigations showed a significantly higher prevalence of HHV-6A/B DNA in both blood and peripheral tissues of SSc patients compared to controls. In particular, the virus was detectable in 80.7% of PBMCs from SSc patients and in only 40% of healthy controls (*p* < 0.001), and the virus load was significantly higher in the SSc group (median copy number 235.5 per 10^6^ cells) compared to healthy subjects (median copy number 18.9 per 10^6^ cells) (*p* < 0.0001). However, the results identified a split population in SSC group, one harboring high virus load and the other with low/no virus, suggesting that HHV-6A/B may contribute to the disease in a subgroup of patients, similarly to what observed in multiple sclerosis [25]. Indeed, at the skin level, the differences were particularly significant; in fact, although the number of skin biopsies available for the analyses was low, five out of five SSc specimens were positive for the presence of HHV-6A/B DNA, whereas no samples derived from healthy skin had detectable amounts of the virus. In the cutaneous biopsies, the species 6A was the most represented, consistent with previously reported data on HHV-6A tropism [52], whereas HHV-6B was present almost exclusively in PBMC, confirming as well previous reports [52,53]. Due to the small dimension of skin biopsies, it was not possible to perform immunochemistry to detect HHV-6A/B antigens, but this could be important in future studies, to verify virus presence.

Importantly, higher virus loads correlated with the severity of scleroderma cutaneous and internal organ involvement, as well as with the presence of worse immunological markers (anti-Scl70 antibodies). In fact, both anti-U94 antibody titer and NK impairment against HHV-6A/B appeared increased in the SSc subgroup displaying high virus load in PBMC and skin. Moreover, the impairment of NK response against the virus was particularly evident and significant in patients expressing KIR2DL2 receptors, as already reported in other autoimmune conditions associated with HHV-6 infection [25], confirming that HHV-6A might express its full pathogenic potential in KIR2DL2 subjects.

Although a similar prevalence of anti-HHV-6A/B antibodies in the SSc group and healthy controls (76.8 vs. 75%), the prevalence and titer of anti-U94 antibodies were significantly higher in the SSc population (100%, median titer 1:400) compared to controls (46.7%, median titer 1:166) (*p* < 0.004), confirming previous similar observations in multiple sclerosis patients [45]. Furthermore, consistently with previous observations showing the ability of virus infection to inhibit angiogenesis by a U94-mediated sHLA-G production in endothelial cells [37], we detected increased levels of sHLA-G in HHV-6A/B positive SSc patients compared to negative ones (*p* < 0.0001). Notably, SSc patients who were both HHV-6A/B and HLA-G positive, also displayed the highest titer of anti-U94 antibodies (*p* < 0.05), supporting a possible role of HHV-6A/B infection in the disease progression.

As proof of concept, we analyzed whether HHV-6 infection could induce pro-fibrotic molecules expression in endothelial cells. Endothelial injury represents, in fact, one of the first steps in the pathogenesis of SSc, predominantly affecting the microcirculation [54]. Aiming to clarify this aspect, HUVEC were infected in vitro with HHV-6A or 6B, and analyzed for the expression of a panel of 84 fibrosis-associated factors. The results showed that HHV-6A and 6B infection induced the upmodulation of several factors, suggesting that infection per se may interfere with the normal functions of endothelial cells [11,37]. Although the TGFβ2 factor, recently recognized as a key factor in fibrosis induction, did not result upregulated by virus infection in HUVECs, other factors belonging to TGFβ superfamily were promoted in particular by HHV-6A, including GREM1 and INHBE, IL-4, IL-5, TNF, and MMP9. Many of such virus-induced factors have been reported as key factors in tissue fibrosis: GREM1 was recognized as a key pro-fibrotic factor in renal, pancreatic, and pulmonary fibrosis [55,56,57]; INHBE, a member of the TGFβ superfamily of proteins, was reported to be associated with dermal fibrosis [58]; IL-4 is even more active than TGFβ in inducing collagen synthesis in human skin fibroblasts [59,60], and can drive fibroblast differentiation and promote pro-fibrotic macrophages activation [61]. IL-5also plays an important role in promoting tissue fibrosis [60,62]; and TNF antagonists are beneficial for the treatment of fibrotic disorders, supporting a pro-fibrotic role of TNF [41,63,64,65]. By contrast, the role of MMP9 is unclear, having a pro-fibrotic or anti-fibrotic action in different rodent models [66,67]. Some anti-fibrotic factors, namely SMAD6, SMAD7, STAT1, and HGF, were also induced by the virus infection, although to a lesser extent compared to pro-fibrotic upregulation, perhaps as a counterbalance of the potent pro-fibrotic expression induced in vitro by virus infection. 

Consistent with our findings, endothelial progenitor cells (EPC) with impaired angiogenic function have been detected in peripheral blood of SSc subjects [68], and the regression of capillaries and small vessels during SSc strongly suggests an impairment in the angiogenetic abilities of endothelial cells, although the specific mechanisms have not yet been elucidated. 

It is conceivable that multiple triggering and predisposing genetic factors can variably contribute to both diffuse microangiopathy and fibrosis observed in SSc, according to the different stages of the disease [69,70,71,72,73] and different SSc subsets [1,3]. In this respect, the observed increased sHLA-G plasma levels and titer of anti-U94 antibodies in SSc subjects might be considered a marker for disease severity and suggest the involvement of the virus and U94 in the development of the disease.

Although several studies provided important information linking infectious agents to SSc, a direct association is still missing. Our virological and immunological data suggest that HHV-6A/B, and in particular the 6A species, might have a role in the disease onset and/or progression and open the way for future studies aimed to confirm these data in a higher number of subjects and focusing on the mechanisms by which viral products might synergize with other factors (82-86), ultimately favoring the SSc development.

## Figures and Tables

**Table 1:** Demographic and clinical features of the study population.

Parameters	SSc Patients	Controls
Number	26	30
Age (median, range)	56 (37–74)	52 (38–65)
*Gender*		
Male (n, %)	4 (15.4%)	5 (16.6%)
Female (n, %)	22 (84.6%)	25 (83.3%)
HHV-6 Ab (n, %)	20 (76.9%)	22 (73.3%)
Disease duration (median years, range)	5 (1–20)	-
mRSS (median, range)	7 (0–33)	-
*Cutaneous subgroups*		
Diffuse cutaneous SSc	5 (19.2%)	-
Limited cutaneous SSc	21 (80.7%)	-
*Clinical signs (n*, *%)*		
Raynaud’s phenomenon	26 (100%)	
Digital ulcer	10 (38.5%)	-
Puffy fingers	14 (53.8%)	-
Pitting	8 (30.7%)	-
Telangiectasia	13 (50%)	-
Arthralgia	13 (50%)	-
Interstitial lung disease	15 (57.7%)	-
Heart involvement	1 (3.8%)	-
Esophageal dysfunction	13 (50%)	-
*Autoantibodies (n*, *%)*		
Positive ANA	25 (96.1%)	-
Positive ACA	9 (34.6%)	-
Positive anti-Scl70	9 (34.6%)	-
*Treatments (n*, %) *		
Steroids	3 (11.5%)	-
Immunosuppressors	3 (11.5%)	-
Prostanoids	19 (73.1%)	-
Bosentan	7 (26.9%)	-
Calcium antagonist	18 (69.2%)	-

mRSS: modified Rodnan skin score; ANA, anti-nuclear autoantibodies; ACA, anti-centromere auto-antibodies; anti-Scl70, anti-topoisomerase type of anti-nuclear antibodies. * Ongoing treatment at the time of patients’ recruitment; ° mycophonolate mofetil.

**Table 2:** Distribution of KIR2-DL2 and KIR2-DL3 types in systemic sclerosis (SSc) patients and healthy subjects.

KIR Type	SSc Patients (*n* = 26)	Controls (*n* = 30)
DL2/DL2	1 (3.8%)	1 (3.3%)
DL2/DL3	8 (30.8%)	11 (33.7%)
DL3/DL3	17 (65.4%)	18 (60%)

## Supplementary Materials

The following are available online at https://www.mdpi.com/2076-2607/8/1/39/s1, Figure S1: Induction of fibrosis-associated factors by HHV-6A/B infection in human endothelial cells (HUVEC).
