# Supplementary figures and images for: HHV-6A Infection and Systemic Sclerosis: Clues of a Possible Association
Source: Microorganisms. 2019 Dec 24;8(1):39. doi: 10.3390/microorganisms8010039 (PMC7022325; doi:10.3390/microorganisms8010039)

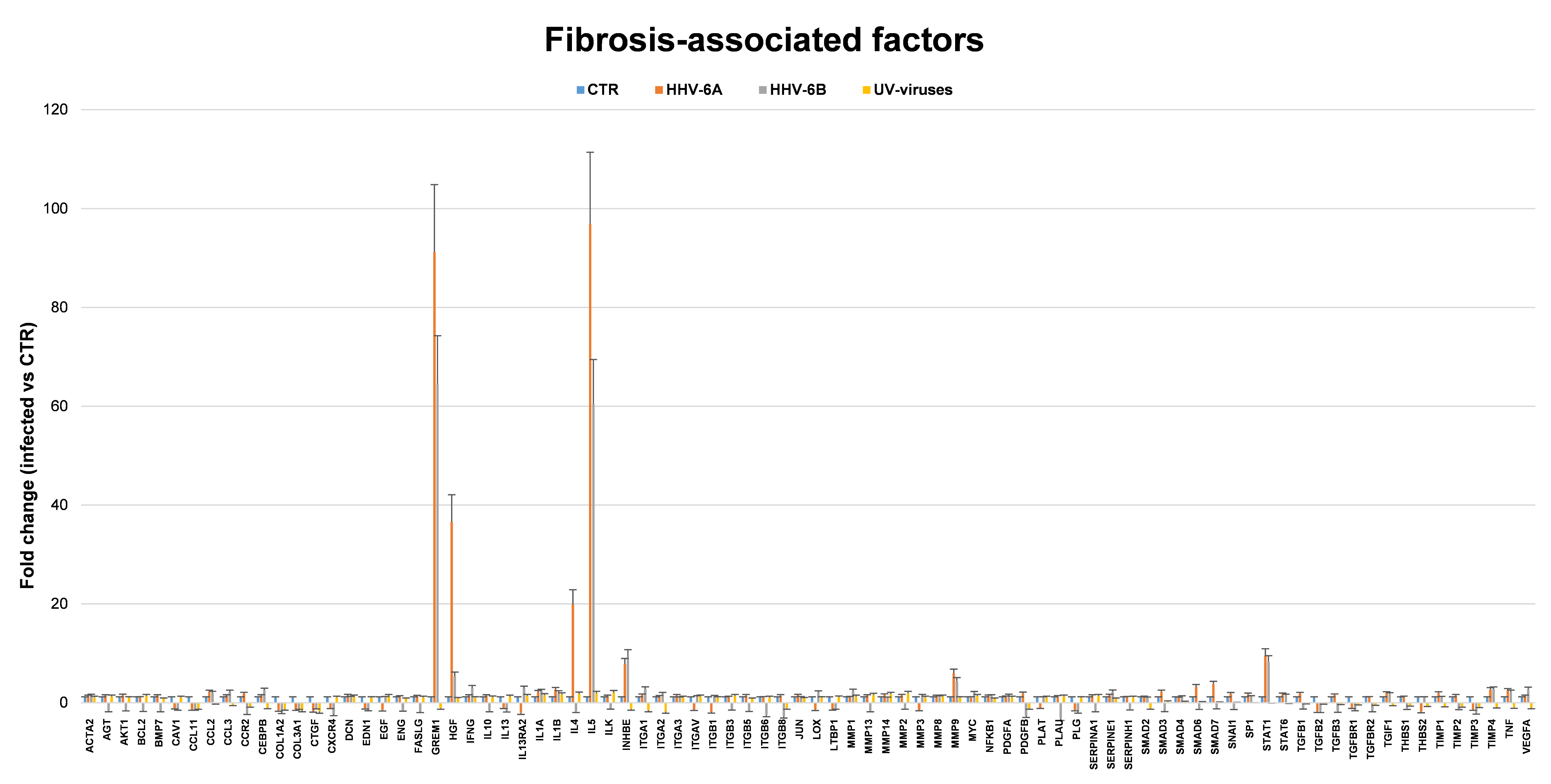

Supplement: Supplementary file 1 [file microorganisms-08-00039-s001.zip › microorganisms-675284-supplementary.tif]
